# Retrospective evaluation of transcranial magnetic stimulation for enhancing arousal in patients with minimally conscious state: a single-centre study in Inner Mongolia, China

**DOI:** 10.3389/fpsyt.2026.1757883

**Published:** 2026-05-05

**Authors:** Wenqi Deng, Sha Wang, Lixia Zhang, Shuqi Wang, Anna Bai, Ziyu Xing, Yu Liang, Yan Zhang, Wenhua Xing, Lixia Hao

**Affiliations:** 1 Department of Rehabilitation Medicine, Inner Mongolia Medical University Affiliated Hospital, Hohhot, China; 2 Department of Rehabilitation Medicine, The Second Affiliated Hospital of Inner Mongolia Medical University, Hohhot, China; 3 Queen Mary College, Nanchang University, Nanchang, China; 4 Department of Spinal Surgery, The Second Affiliated Hospital of Inner Mongolia Medical University, Hohhot, China

**Keywords:** arousal promotion, disorders of consciousness, minimally conscious state, rehabilitation, transcranial magnetic stimulation

## Abstract

**Objective:**

This study aimed to investigate the effect of high-frequency transcranial magnetic stimulation (TMS) targeting multiple brain regions on the recovery of consciousness in patients with minimally conscious state (MCS).

**Methods:**

A retrospective analysis was conducted on MCS patients between August 2022 and March 2024. Some patients received only conventional rehabilitation treatment, while others received additional TMS therapy. Clinical outcomes were assessed using the Glasgow Coma Scale (GCS) and the Coma Recovery Scale-Revised (CRS-R) at three time points: before treatment (T0), two weeks post-treatment (T1), and one month post-treatment (T2). Additional assessments included electroencephalogram (EEG), brainstem auditory evoked potential (BAEP), somatosensory evoked potentials (SEP), and serum levels of brain-derived neurotrophic factor (BDNF) and neuron-specific enolase (NSE).

**Results:**

A total of 30 patients were included and divided equally into two groups. The GCS and CRS-R scores of the 15 patients who received TMS therapy demonstrated significant improvements at T1 and T2. Furthermore, these patients exhibited significant enhancements in EEG and BAEP grading at T1.

**Conclusion:**

The findings suggest that adjunctive multi-target high-frequency repetitive TMS may promote recovery of consciousness in MCS patients. These results underscore the potential of repetitive TMS as a therapeutic intervention for MCS and warrant further investigation in future studies.

## Introduction

Advances in modern medicine and critical care have significantly improved the survival of patients with severe brain injuries, as well as those with conditions such as stroke and brain tumours, however, this progress has led to a substantial increase in the number of patients with disorders of consciousness (DoC) (1, 2). Epidemiological data indicate that approximately 100,000 to 300,000 individuals in the United States are affected by DoC (3), and 0.2 to 6.1 cases per 100,000 individuals are reported in Europe (4). In China, more than 100,000 new cases of DoC are diagnosed annually, with an existing patient population estimated at 70,000 to 100,000. Each year, thousands of patients remain in a long-term minimally conscious state (MCS) (5). The annual cumulative medical expenditure associated with these cases is approximately 30 to 50 billion yuan, imposing a substantial burden on both families and the national healthcare system (6). Despite advances in neurocritical care, effective therapeutic strategies to promote recovery of consciousness remain limited. Accordingly, exploring non-invasive neuromodulation approaches, such as repetitive transcranial magnetic stimulation (rTMS), is of considerable clinical importance.

Current non-invasive treatments for DoC primarily include transcranial direct current stimulation (tDCS). Conventional tDCS delivers current via a pad electrode; it does not directly induce neuronal activity but can alter membrane potentials (7). The left dorsolateral prefrontal cortex (DLPFC) is the most commonly targeted region (8). Newer techniques, including transcranial ultrasonic stimulation (TUS) and transcutaneous auricular vagus nerve stimulation (taVNS), have also advanced considerably in recent years (9). Beyond brain stimulation, systemic therapies such as hyperbaric oxygen therapy (10) and acupuncture (11) have demonstrated efficacy in the management of DoC. Although these approaches show promise, further validation of optimised stimulation parameters, alternative pharmacological interventions, and rehabilitation strategies is required to improve rehabilitation outcomes and enhance quality of life for patients (12).

Transcranial magnetic stimulation (TMS) is a non-invasive neuromodulation technique developed in recent years (13). It has shown considerable potential in treating a variety of neuropsychiatric disorders, including chronic pain, depression, Parkinson’s disease, and stroke (14). Studies have demonstrated that TMS exerts arousal-promoting effects in patients with DoC, particularly those in MCS, where it has shown significant clinical efficacy (15). Research indicates that rTMS modulates distal brain networks by precisely targeting specific cortical regions (16), rTMS applies finely modulated time-varying magnetic and electric fields to evoke neuronal action potentials, which are then transmitted to other cortical regions via synaptic connections (17). For example, one study reported that TMS applied to the dorsal premotor cortex induced excitatory modulation in the primary motor cortex (M1) in DoC patients (18), while another explored its use in arousal-promoting therapies for DoC (19). Neuroimaging evidence further indicates changes in blood oxygen level-dependent (BOLD) signals (20) and cerebral blood flow (CBF) (21) in DoC patients following rTMS targeting the premotor or motor cortex. Notably, patients in MCS demonstrate significant increases in CBF.

Based on these considerations, this study aimed to evaluate the effects of a novel treatment approach applying rTMS sequentially to the DLPFC and M1 in patients with MCS. We hypothesised that high-frequency multi-target rTMS applied to bilateral DLPFC and M1 would enhance cortical excitability, improve large-scale network connectivity, and promote behavioural recovery in patients with MCS compared with conventional rehabilitation alone. Specifically, we anticipated that this combined stimulation would produce synergistic effects by simultaneously engaging top-down cognitive control networks and bottom-up arousal pathways, thereby facilitating the reintegration of consciousness-related neural circuits.

## Methods

### Study design and patients

This retrospective study included patients with MCS who were admitted to the Rehabilitation Department of the Inner Mongolia Medical University Affiliated Hospital between August 2022 and March 2024. Medical records of potential participants were reviewed to determine eligibility. The inclusion criteria were: 1) diagnosis of MCS according to the 2020 European Diagnostic Guidelines for Coma and Mental Disorders (22); 2) age between 18 and 80 years; 3) initiation of rehabilitation treatment within 1 to 6 months following diagnosis; and 4) implementation of standardised clinical behavioural assessment scales, with rigorous screening prior to EEG data collection. Exclusion criteria were: 1) incomplete data; 2) failure to complete rehabilitation treatment; 3) use of medications known to significantly affect consciousness, such as sedatives, hypnotics, or antiepileptic drugs, within 24 hours prior to data collection; and 4) presence of unstable vital signs or concurrent conditions, including severe infection, deep vein thrombosis in the lower limbs, or severe organ failure during treatment. This study was approved by the Ethics Committee of Inner Mongolia Medical University Affiliated Hospital (Approval Number: YJS2024034). Due to the retrospective design and the use of anonymized clinical data, no additional informed consent for participation in this study was required.

All patients received standard rehabilitation, which included hyperbaric oxygen therapy, acupuncture, physical rehabilitation, and pharmacological treatment. In addition to this standard protocol, some patients received adjunctive magnetic stimulation therapy targeting the bilateral DLPFC and bilateral primary motor cortex. As part of routine clinical care, patients or their families were informed about the potential benefits, risks, and costs of the intervention. The decision to receive additional TMS therapy was made based on routine clinical practice and patient or family preference. For the magnetic stimulation therapy, the stimulation intensity was set at 100% of the resting motor threshold, with a frequency of 10 Hz and a 10-second interval between pulse trains. Treatment was administered once daily, delivering 2000 pulses per session, five times per week, over two consecutive weeks.

### Outcome and data collection

The primary outcomes of this study were neurobehavioral evaluations and neuroelectrophysiological indicators. Neurobehavioral assessments were conducted at three time points: prior to the initiation of rTMS treatment (T0, baseline), at the completion of treatment (T1, two weeks post-treatment), and at follow-up (T2, one month after the end of treatment). The Glasgow Coma Scale (GCS) (23) and Coma Recovery Scale-Revised (CRS-R) (24) were applied to all patients. CRS-R scores were independently assessed five times at different time points and in various states, and the highest score was selected as the final CRS-R result. Neurobehavioral evaluations were performed by the same physician with 17 years of experience in rehabilitation medicine. Neuroelectrophysiological indicators included electroencephalogram (EEG), brainstem auditory evoked potential (BAEP), and somatosensory evoked potentials (SEP).

EEG assessments were tailored according to individual patient conditions and graded using the Synek system (25). Level I is dominated by regular alpha waves with minor theta activity; Level II is dominated by theta waves, with amplitudes within or below the normal range; Level III primarily exhibits prominent delta and spindle wave activity; Level IV shows markedly abnormal rhythmic features, including diffuse delta waves and short-range electrical silence; Level V reflects a completely suppressed state, with electrical silence below 0.2 µV.

BAEP and SEP were classified using Greenberg’s standardised system (26). For BAEP: Level I indicates a normal response; Level II allows identification of waves I-V, but with prolonged latencies and reduced amplitudes; Level III shows normal latency and amplitude for wave I, while other waves are unclear or difficult to identify; Level IV reflects poor waveform resolution, with only wave I observable. For SEP: Level I represents a normal physiological state; Level II indicates acceptable N20 differentiation, but with prolonged latency and reduced amplitude; Level III exhibits poor N20 differentiation, though the waveform remains recognisable; Level IV represents the disappearance of all waveforms, indicating complete abnormality.

Laboratory indicators included venous blood samples collected at T0 and T1. Serum levels of brain-derived neurotrophic factor (BDNF) and neuron-specific enolase (NSE) were measured using enzyme-linked immunosorbent assay (ELISA). Functional neuroimaging data were acquired using Diffusion Tensor Imaging (DTI) and three-dimensional Arterial Spin Labelling (3D-ASL). DTI was employed to track nerve fibre bundles in consciousness-related brain regions and to assess changes in brain network connectivity in patients with disorders of consciousness by generating fractional anisotropy (FA) maps using 3D Slicer software before and after TMS treatment. The 3D-ASL technique was used to observe changes in CBF in individual patients pre- and post-treatment.

### Statistical methods

Statistical analyses were performed using SPSS version 26.0 (SPSS Inc., Chicago, USA). Normality was assessed using the Shapiro-Wilk test. Continuous data with a normal distribution were presented as mean ± standard deviation (SD) and compared using Student’s t-test; non-normally distributed data were expressed as median (interquartile range, IQR) and analysed using the Wilcoxon rank-sum test. Categorical data were presented as n (%) and analysed using the chi-square test. For pairwise comparisons with significant results in analyses of variance, the Bonferroni correction was applied. Two-sided P-values <0.05 were considered statistically significant.

## Result

### Basic characteristics of the participants

A total of 30 patients with MCS were included in the study, comprising 15 patients who received magnetic stimulation therapy and 15 patients who received only standard rehabilitation treatment. There were no significant differences between the groups in terms of age (P = 0.277), gender (P = 0.713), or aetiology (P = 0.317) (**Table 1**).

**Table 1:** Basic characteristics of the participants.

Variable	Total (n=30)	Magnetic stimulation (n=15)	Without magnetic stimulation (n=15)	P value
Gender, n (%)				0.713
Male	17 (56.7)	9 (60.0)	8 (53.3)	
Female	13 (43.3)	6 (40.0)	7 (46.7)	
Age/Year	57.90 ± 12.99	55.27 ± 16.06	60.53 ± 8.75	0.277
Pathogeny, n (%)				0.317
Cerebral haemorrhage	16 (53.3)	9 (60.0)	7 (46.7)	
Cerebral infarction	3 (20.0)	0 (0.0)	3 (20.0)	
Brain trauma	5 (33.3)	6 (40.0)	5 (33.3)	

### Effect of rTMS on GCS score

Intra-group comparisons showed that in patients who did not receive magnetic stimulation therapy, both the GCS total score and the motor response score at T1 and T2 were significantly higher than at T0 (P < 0.05). In patients receiving magnetic stimulation therapy, the GCS total score, eye-opening response score, verbal response score, and motor response score were all significantly improved at T1 and T2 compared with T0 (P < 0.05). Furthermore, in this group, the GCS total score and motor response score at T2 were significantly higher than at T1 (P < 0.05).

Inter-group comparisons indicated no significant difference in the total GCS score at T0 between patients who received magnetic stimulation therapy and those who did not (7.67 ± 1.05 vs. 7.87 ± 1.51, P = 0.676). At T1, patients receiving magnetic stimulation therapy showed greater improvements in total GCS score (9.73 ± 1.16 vs. 8.67 ± 0.98, P = 0.011), eye-opening score (3.87 ± 0.35 vs. 3.33 ± 0.72, P = 0.018), and verbal response score (1.80 ± 0.68 vs. 1.07 ± 0.26, P < 0.001). At T2, the same group continued to exhibit higher total GCS scores (P = 0.001), eye-opening scores (3.87 ± 0.35 vs. 3.33 ± 0.82, P = 0.031), and verbal response scores (2.27 ± 1.10 vs. 1.13 ± 0.52, P = 0.001) compared with patients who did not receive magnetic stimulation therapy (**Table 2**).

**Table 2:** Comparison of GCS indicators.

Variable	Total population (n=30)	Without magnetic stimulation (n=15)	Magnetic stimulation (n=15)	P value
T0				
total	7.77 ± 1.28	7.67 ± 1.05	7.87 ± 1.51	0.676
Eye opening	3.20 ± 0.76	3.07 ± 0.80	3.33 ± 0.72	0.346
Verbal response	1.13 ± 0.35	1.00 ± 0.00	1.27 ± 0.46	**0.041**
Motor response	3.40 ± 1.10	3.60 ± 0.83	3.20 ± 1.32	0.329
T1				
total	9.20 ± 1.19	8.67 ± 0.98^1^	9.73 ± 1.16^1^	**0.011**
Eye opening	3.60 ± 0.62	3.33 ± 0.72	3.87 ± 0.35^1^	**0.018**
Verbal response	1.43 ± 0.63	1.07 ± 0.26	1.80 ± 0.68^1^	**<0.001**
Motor response	4.17 ± 0.79	4.27 ± 0.80^1^	4.07 ± 0.80^1^	0.499
T2				
total	9.90 ± 2.09	8.73 ± 1.53^1^	11.07 ± 1.94^1,2^	**0.001**
Eye opening	3.60 ± 0.67	3.33 ± 0.82	3.87 ± 0.35^1^	**0.031**
Verbal response	1.70 ± 1.02	1.13 ± 0.52	2.27 ± 1.10^1^	**0.001**
Motor response	4.60 ± 1.07	4.27 ± 0.96^1^	4.93 ± 1.10^1,2^	0.088

^1^ Compared with T0, *P* < 0.05; ^2^Compared with T1, *P* < 0.05. Bold values indicate statistical significance (*P* < 0.05).

### Effects of rTMS on CRS-R scores

Intra-group comparisons demonstrated that in patients who did not receive magnetic stimulation therapy, the CRS-R total score, auditory score, visual score, and motor score were significantly higher at T1 and T2 than at T0 (P < 0.05). In patients receiving magnetic stimulation therapy, all scores except the communication score were significantly improved at T1 compared with T0 (P < 0.05), and all observation scores were significantly improved at T2 (P < 0.05). Compared with T1, the total CRS-R score, auditory score, visual score, and motor score were significantly higher at T2 (P < 0.05).

Inter-group comparisons showed no statistically significant difference in total CRS-R scores at T0 between patients who received magnetic stimulation therapy and those who did not (7.80 ± 1.21 vs. 8.93 ± 1.79, P = 0.052). However, patients receiving magnetic stimulation therapy had significantly higher communication scores (0.60 ± 0.74 vs. 0.00 ± 0.00, P = 0.007). At T1, this group demonstrated higher total CRS-R scores (13.07 ± 2.74 vs. 9.87 ± 2.90, P = 0.004), auditory scores (2.60 ± 0.51 vs. 1.93 ± 0.59, P = 0.003), communication scores (1.33 ± 0.98 vs. 0.07 ± 0.26, P < 0.001), and arousal scores (2.53 ± 0.52 vs. 2.13 ± 0.52, P = 0.043) compared with patients who did not receive magnetic stimulation therapy. At T2, all observation scores except the motor score (P = 0.116) were significantly higher in patients receiving magnetic stimulation therapy (all P < 0.01) (**Table 3**).

**Table 3:** Comparison of CRS-R indicators.

Variable	Total population (n=30)	Without magnetic stimulation (n=15)	Magnetic stimulation (n=15)	P value
T0				
total	8.37 ± 1.61	7.80 ± 1.21	8.93 ± 1.79	0.052
aural	1.60 ± 0.62	1.53 ± 0.52	1.67 ± 0.72	0.566
visually	1.90 ± 0.66	1.67 ± 0.62	2.13 ± 0.64	0.052
motion	2.63 ± 0.72	2.87 ± 0.35	2.40 ± 0.91	0.075
spoken	0.30 ± 0.60	0.00 ± 0.00	0.60 ± 0.74	**0.007**
interaction	0.10 ± 0.31	0.00 ± 0.00	0.20 ± 0.41	0.072
awakened	1.87 ± 0.63	1.80 ± 0.68	1.93 ± 0.59	0.571
T1				
total	11.47 ± 3.21	9.87 ± 2.90^1^	13.07 ± 2.74^1^	**0.004**
aural	2.27 ± 0.64	1.93 ± 0.59^1^	2.60 ± 0.51^1^	**0.003**
visually	2.40 ± 0.86	2.13 ± 1.06^1^	2.67 ± 0.49^1^	0.092
motion	3.53 ± 0.94	3.53 ± 0.83^1^	3.53 ± 1.06^1^	1.000
spoken	0.70 ± 0.95	0.07 ± 0.26	1.33 ± 0.98^1^	**<0.001**
interaction	0.23 ± 0.50	0.07 ± 0.26	0.40 ± 0.63	0.069
awakened	2.33 ± 0.55	2.13 ± 0.52	2.53 ± 0.52^1^	**0.043**
T2				
total	13.30 ± 4.36	10.40 ± 2.85^1^	16.20 ± 3.65^1,2^	**<0.001**
aural	2.53 ± 0.78	2.00 ± 0.53^1^	3.07 ± 0.59^1,2^	**<0.001**
visually	2.73 ± 1.11	2.20 ± 1.01^1^	3.27 ± 0.96^1,2^	**0.006**
motion	4.23 ± 1.04	3.93 ± 0.88^1^	4.53 ± 1.13^1,2^	0.116
spoken	0.87 ± 1.11	0.07 ± 0.26	1.67 ± 1.05^1^	**<0.001**
interaction	0.43 ± 0.73	0.07 ± 0.26	0.80 ± 0.86^1^	**0.006**
awakened	2.50 ± 0.57	2.13 ± 0.52	2.87 ± 0.35^1^	**<0.001**

^1^ Compared with T0, *P* < 0.05; ^2^Compared with T1, *P* < 0.05. Bold values indicate statistical significance (*P* <  0.05).

### Comparison of EEG grading

Intra-group analysis showed no significant change in EEG grading among patients receiving standard rehabilitation treatment between pre-treatment (T0) and post-treatment (T1) (P = 0.326). In contrast, patients receiving magnetic stimulation therapy demonstrated a significant improvement in EEG grading from T0 to T1 (P < 0.001). Inter-group analysis revealed no significant difference in EEG grading at T0 between patients who received magnetic stimulation therapy and those who did not (P = 0.317). However, at T1, EEG grading was significantly higher in the magnetic stimulation therapy group compared with the standard rehabilitation group (P < 0.001) (**Table 4**).

**Table 4:** Comparison of EEG, BAEP and SEP grades before and after treatment.

Examination	Time/grade	Magnetic stimulation	Without magnetic stimulation	P value	P value (Within MS)	P value (Without MS)
EEG grade	T0			0.317		
	Grade I	0	0			
	Grade II	0	0			
	Grade III	0	0			
	Grade IV	15	14			
	Grade V	0	1			
	T1			<0.001	<0.001	0.326
	Grade I	13	1			
	Grade II	0	1			
	Grade III	0	0			
	Grade IV	2	12			
	Grade V	0	1			
BAEP	T0			0.875		
	Grade I	2	1			
	Grade II	2	5			
	Grade III	9	6			
	Grade IV	2	3			
	T1			0.104	0.016	0.618
	Grade I	5	3			
	Grade II	7	4			
	Grade III	2	5			
	Grade IV	1	3			
SEP	T0			0.395		
	Grade I	0	0			
	Grade II	0	0			
	Grade III	0	0			
	Grade IV	15	14			
	Grade V	0	1			
	T1			0.208	<0.001	0.312
	Grade I	13	1			
	Grade II	0	1			
	Grade III	0	0			
	Grade IV	2	12			
	Grade V	0	1			

EEG, electroencephalogram; BAEP, brainstem auditory evoked potential; SEP, somatosensory evoked potentials; MS, Magnetic Stimulation.

### Comparison of BAEP grading

Intra-group analysis showed no significant difference in BAEP grading from T0 to T1 among patients receiving standard rehabilitation alone (P = 0.618). Conversely, patients receiving magnetic stimulation therapy exhibited a significant improvement in BAEP grading from T0 to T1 (P = 0.016). Inter-group analysis indicated no significant difference in BAEP grading between the two groups at T0 (P = 0.875) or at T1 (P = 0.104) (**Table 4**).

### Comparison of SEP grading

Intra-group analysis showed no significant difference in SEP grading from T0 to T1 in either the magnetic stimulation therapy group or the standard rehabilitation group (all P > 0.05). Inter-group comparisons similarly revealed no significant differences at T0 (P = 0.395) or T1 (P = 0.208) (**Table 4**).

### Comparison of BDNF and NSE

Intra-group comparisons demonstrated that BDNF levels decreased significantly from T0 to T1 in both patients receiving magnetic stimulation therapy and those receiving standard rehabilitation alone (P < 0.001). Inter-group analysis showed no significant difference in BDNF levels at T0 (P > 0.05). At T1, however, patients receiving magnetic stimulation therapy exhibited significantly lower BDNF levels than those receiving standard rehabilitation alone (P = 0.016) (**Supplementary Figure 1**). Similarly, NSE levels decreased significantly from T0 to T1 in both groups (P < 0.001).Inter-group comparisons showed no significant difference in NSE levels at T0 (P > 0.05), whereas at T1, patients receiving magnetic stimulation therapy had significantly lower NSE levels than those not receiving the therapy (P = 0.003) (**Supplementary Figure 2**).

### Comparison of CBF maps of in a MCS patients before and after TMS treatment

3D-ASL perfusion imaging in patients receiving magnetic stimulation therapy revealed decreasedCBF in the basal ganglia at T0. Following treatment (T1), CBF in the basal ganglia increased relative to pre-treatment levels. In contrast, patients receiving standard rehabilitation alone showed no notable changes in CBF before and after treatment (**Supplementary Figure 3**, **Table 5**).

**Table 5:** General clinical information of patients with cerebral blood flow images.

Patient	Sex	Course of disease/d	Cause of disease	Age (years)	Time	Consciousness
P1	male	66	traumatic brain injury	38	T0	MCS-
					T1	MCS-
P3	female	18	traumatic brain injury	61	T0	MCS-
					T1	MCS-
P6	female	45	cerebral haemorrhage	63	T0	MCS-
					T1	MCS-
P7	male	25	cerebral haemorrhage	74	T0	MCS-
					T1	MCS-
P16	male	60	cerebral haemorrhage	59	T0	MCS-
					T1	MCS+
P18	female	40	cerebral infarction	64	T0	MCS-
					T1	MCS+
P21	female	20	cerebral infarction	71	T0	MCS-
					T1	MCS+
P22	male	27	traumatic brain injury	40	T0	MCS+
					T1	MCS+

### Nerve fibre tractography of MCS patients before and after TMS treatment

At T0, DTI tractography in patients receiving magnetic stimulation therapy showed sparse peripheral nerve fibres in the medulla oblongata (**Supplementary Figure 4A**). After treatment (T1), there was an apparent increase in fibre density around the brainstemmedulla (**Supplementary Figure 4B**). Similarly, DTI tracking of fibres surrounding the thalamus revealed sparse bundles at T0(**Supplementary Figure 4C**), which increased following TMS treatment at T1 (**Supplementary Figure 4D**).

## Discussion

This study demonstrated that the combination of multi-target high-frequency rTMS with conventional rehabilitation was superior to conventional rehabilitation alone in improving consciousness in patients with MCS. Following rTMS treatment, significant improvements were observed in GCS scores, CRS-R scores, and EEG grading. Additionally, BDNF and NSE levels showed a more pronounced decrease in patients who received magnetic stimulation therapy. DTI and CBF findings also indicated increased perfusion and enhanced connectivity in patients receiving magnetic stimulation therapy.

The theoretical rationale for selecting bilateral DLPFC and M1 as stimulation targets lies in their hierarchical complementarity within the consciousness network. The DLPFC, as a core node of the frontoparietal network, plays a critical role in higher-order regulation of consciousness, including attentional control and the updating of conscious content. High-frequency rTMS applied to the DLPFC has been shown to significantly improve the level of consciousness in patients with MCS by modulating dynamic interactions among the frontoparietal network, default mode network, and salience network (27). The M1 is not merely a motor output region but also an important node within the thalamocortical circuitry and the ascending reticular activating system, contributing to arousal maintenance and sensorimotor integration. Stimulation of M1 can activate thalamocortical pathways in a retrograde manner and enhance overall cortical excitability (28). Therefore, simultaneous targeting of these regions may produce a synergistic effect by engaging both top-down cognitive control and bottom-up arousal mechanisms. This dual modulation may facilitate the re-establishment of the critical network balance required for the recovery of consciousness, which is consistent with the findings of the present study. Although direct clinical evidence supporting this synergistic effect remains limited, the strategy is physiologically plausible and warrants further investigation.

In the present study, patients receiving transcranial magnetic stimulation therapy exhibited greater improvements in CRS-R scores compared with those reported in previous studies (an average increase of 4.14 points at T1 and 7.27 points at T2). For example, Xia et al. reported that patients receiving single-target rTMS stimulation of the DLPFC showed an average improvement of approximately 2.6 points in CRS-R scores (29). These findings further suggest that multi-target stimulation strategies may be more effective in improving consciousness than single-target approaches.

One of the key factors influencing the efficacy of rTMS in promoting arousal is the duration of stimulation. Previous studies have demonstrated a cumulative effect of rTMS in enhancing arousal (30), and prolonged stimulation protocols are therefore commonly employed. In this study, rTMS was administered once daily for 40 minutes per session, for a total of 10 sessions at a frequency of 10 Hz. The results showed significant improvements in GCS and CRS-R scores, indicating enhanced levels of consciousness in patients with MCS. Previous research has also indicated that prolonged rTMS not only produces immediate therapeutic effects but may induce sustained aftereffects lasting for at least seven days (31). In the present study, follow-up assessments conducted one month after treatment demonstrated further improvements in neurobehavioural scores among patients who received magnetic stimulation therapy. These findings highlight the importance of further investigating prolonged rTMS protocols for arousal promotion.

In recent years, advances in neuroelectrophysiology have provided clinicians with more comprehensive insights into brain function, thereby improving the accuracy and reliability of diagnosing and evaluating DoC. EEG in patients with DoC typically demonstrates diffuse theta (θ) and delta (δ) activity. Detailed analysis of EEG background activity can provide an initial assessment of the level of consciousness, such as distinguishing between a vegetative state and MCS, and is therefore of considerable importance in evaluating consciousness levels and identifying abnormalities in brain function (32).

BAEP is employed to assess the degree of brainstem dysfunction. By recording neuroelectric changes induced by auditory stimuli, BAEP reflects the integrity of the auditory conduction pathways and brainstem neurons. When brainstem injury occurs, alterations in BAEP waveforms and latencies may provide insight into the extent and nature of the damage (33). Notably, bilateral V waves are closely associated with the functional state of the ascending reticular activating system.

Somatosensory evoked potentials (SEP), particularly the N20 wave, are critical indicators for evaluating the functional status of cortical and sensory conduction pathways. Analysis of the N20 wave provides valuable insight into neural activity within key brain regions, facilitating a more accurate understanding of individual sensory and cognitive functions.

Studies have demonstrated that high-frequency repetitive rTMS effectively enhances the presence of theta (θ), alpha (α), and delta (δ) waves in EEG recordings of DoC patients (30), while also improving BAEP V wave latency and I-V interwave latency (34). The findings of the present study similarly indicate that rTMS treatment led to improvements in EEG and BAEP grading among patients who received magnetic stimulation therapy. These results suggest that high-frequency rTMS effectively modulates cortical activity in patients with MCS, improves the functionality of the ascending reticular activating system in the brainstem, and promotes the normalisation of subcortical conduction pathways.

Furthermore, the application of 10 Hz multi-target rTMS was found to reduce slow-wave discharges, enhance alpha rhythms, and increase cortical excitability. It also ameliorated suppressed states of the ascending reticular activating system in the brainstem and subcortical conduction pathways, thereby facilitating self-repair mechanisms in damaged neurons. These outcomes underscore the significant role of high-frequency rTMS in improving consciousness states and neuroelectrophysiological function in patients with MCS.

Following rTMS treatment in patients with DoC, significant changes in serum biomarkers, such as BDNF and NSE, have been observed. In this study, compared with pre-treatment levels, serum BDNF and NSE levels were significantly reduced, regardless of whether patients received magnetic stimulation therapy. Moreover, patients who received rTMS demonstrated greater efficacy in improving neurobehavioural scores and reducing serum BDNF and NSE levels. Interestingly, serum BDNF levels decreased in both groups after treatment, which contrasts with some previous studies reporting increased BDNF following neuromodulation. This discrepancy may be attributed to differences in disease stage, sampling time, or genetic polymorphisms in the BDNF gene (35, 36). In addition, in the early stages of the disease, peripheral BDNF levels are influenced by stress states and systemic inflammatory responses, leading to a significant increase in serum levels that gradually normalise over time (37, 38).

The reduction in BDNF levels may be attributed to the magnetic and electrical responses induced by TMS, which could affect serum BDNF concentrations. One possible explanation is that TMS promotes the utilisation and uptake of BDNF by the central nervous system, leading to a temporary decrease in peripheral BDNF levels. Previous studies have shown that peripheral blood BDNF levels do not directly correspond to BDNF expression in the central nervous system, and a dynamic redistribution process may exist between the two compartments (39). In addition, BDNF expression exhibits periodic changes, which may increase transiently during the early stages of neural regulation and subsequently stabilise or even decrease during network remodelling (40). Therefore, the observed decrease in BDNF in this study may reflect dynamic regulation during neural network reconstruction rather than a reduction in neural plasticity. In summary, further prospective studies with larger sample sizes and parallel cerebrospinal fluid analyses are required to elucidate the role of BDNF in consciousness recovery based on the findings of this study. Similarly, the reduction in NSE levels may be associated with rTMS-mediated modulation of excitatory and inhibitory neurotransmitter concentrations, enhancing synaptic plasticity and thereby leading to decreased serum NSE levels.

Neuroimaging plays a critical role in the evaluation of DoC. DTI is currently one of the most effective techniques for visualising fibre tracts (41), while 3D-ASL enables the assessment of cerebral blood flow changes (42). Studies indicate that rTMS can activate specific cortical regions and their functionally related remote areas, thereby facilitating cortical functional remodelling in various diseases, including Alzheimer’s disease (43) and stroke (44). Importantly, the biological effects of rTMS persist for a period after stimulation has ceased, contributing to the reorganisation of overall neural networks and the restoration of information transmission pathways.

In this study, DTI-based fibre tractography was performed in a subset of patients before and after rTMS. The results demonstrated an increase in fibre bundles within the brainstem and thalamic regions following rTMS. In addition, 3D-ASL monitoring indicated that rTMS enhanced cerebral perfusion in patients with DoC, which may partially contribute to the promotion of consciousness recovery. However, the significant increase in fibre bundle density observed on DTI after two weeks of rTMS treatment should be interpreted with caution. Structural white matter remodelling is generally considered to occur over longer time scales, and short-term changes may reflect alterations in diffusion characteristics, resolution of oedema, or methodological variability, rather than true axonal regeneration. Therefore, these imaging findings should be regarded as preliminary and exploratory, and longitudinal studies based on quantitative tract-based spatial statistics are warranted in the future.

Taken together, the multimodal findings of this study suggest a potential mechanistic framework for rTMS-induced recovery of consciousness. High-frequency multi-target stimulation may enhance cortical excitability (reflected by EEG improvements), strengthen thalamocortical and brainstem connectivity (reflected by BAEP changes and DTI findings), and improve cerebral perfusion (observed in ASL imaging). These neurophysiological and haemodynamic changes may collectively facilitate large-scale network reintegration, which is considered fundamental for the restoration of conscious awareness. Although causality cannot be established in this retrospective study, the convergence of behavioural, electrophysiological, biochemical, and imaging findings provides preliminary support for a network-based mechanism of consciousness recovery.

However, several limitations should be acknowledged. First, this was a single-centre retrospective study with a limited sample size, and the conclusions require validation in large-scale prospective cohorts before clinical application. In addition, more advanced statistical analyses, such as multivariable regression or mediation analysis, were not performed owing to the limited sample size, which may restrict the ability to fully control for potential confounding factors. Second, no blinding of participants or outcome assessors was implemented due to the retrospective design. Furthermore, no formal sample size calculation was conducted prior to the study, which may limit the statistical power of the analysis. Treatment allocation was determined by clinical decision-making at the time of hospitalisation and by patient and family preferences; therefore, sham TMS stimulation was not employed. Moreover, not all patients underwent functional neuroimaging examinations, and only a small number of cases were included in the results. Finally, the relationships among biomarkers, clinical indicators, and neuroimaging parameters require further investigation.

## Conclusion

This study demonstrated that the combination of multi-target high-frequency rTMS and conventional rehabilitation significantly outperformed conventional rehabilitation alone in improving consciousness in patients with MCS. These findings suggest that a multi-target high-frequency rTMS protocol may facilitate arousal and recovery in patients with MCS. In addition, the integrated assessment of behavioural, neurophysiological, laboratory, and imaging indicators provides preliminary support for investigating the neuropathological mechanisms underlying brain network dysfunction in patients with disorders of consciousness.

## Data Availability

The original contributions presented in the study are included in the article/**Supplementary Material**. Further inquiries can be directed to the corresponding authors.

## References

[1] Markov NT Lindbergh CA Staffaroni AM Perez K Stevens M Nguyen K . Age-related brain atrophy is not a homogenous process: different functional brain networks associate differentially with aging and blood factors. Proc Natl Acad Sci USA. (2022) 119:e2207181119. doi: 10.1073/pnas.2207181119. PMID: 36459652 PMC9894212

[2] Snider SB Temkin NR Barber J Edlow BL Giacino JT Hammond FM . Predicting functional dependency in patients with disorders of consciousness: a TBI-model systems and TRACK-TBI study. Ann Neurol. (2023) 94:1008–23. doi: 10.1002/ana.26741. PMID: 37470289 PMC10799195

[3] Giacino JT Katz DI Schiff ND Whyte J Ashman EJ Ashwal S . Comprehensive systematic review update summary: disorders of consciousness: report of the guideline development, dissemination, and implementation subcommittee of the American Academy of Neurology; the American Congress of Rehabilitation Medicine; and the National Institute on Disability, Independent Living, and Rehabilitation Research. Arch Phys Med Rehabil. (2018) 99:1710–9. doi: 10.1016/j.apmr.2018.07.002. PMID: 30098792

[4] Van Erp WS Lavrijsen JC Van De Laar FA Vos PE Laureys S Koopmans RT . The vegetative state/unresponsive wakefulness syndrome: a systematic review of prevalence studies. Eur J Neurol. (2014) 21:1361–8. doi: 10.1111/ene.12483. PMID: 25039901

[5] Bodien YG Busl KM Chang CWJ Claassen J Gaspard N Gosseries O . Disorders of consciousness diagnosis, interventions, and prognostication for the intensivist: Report of the 2025 ISICEM roundtable. Intensive Care Med. (2026) 52:42–62. doi: 10.1007/s00134-025-08224-1. PMID: 41396553 PMC12852174

[6] Zhao J . Disorders of consciousness in China. Neurosci Bull. (2018) 34:605–14. doi: 10.1007/s12264-018-0263-1. PMID: 30039244 PMC6060217

[7] Mensen A Bodart O Thibaut A Wannez S Annen J Laureys S . Decreased evoked slow-activity after tDCS in disorders of consciousness. Front Syst Neurosci. (2020) 14:62. doi: 10.3389/fnsys.2020.00062. PMID: 33100977 PMC7546425

[8] Zhang Y Chen W Zhang T Du J Li R Huo R . P300 correlates with tDCS response in minimally conscious state patients. Neurosci Lett. (2022) 774:136534. doi: 10.1016/j.neulet.2022.136534. PMID: 35181480

[9] Wan X Zhang Y Li Y Song W . An update on noninvasive neuromodulation in the treatment of patients with prolonged disorders of consciousness. CNS Neurosci Ther. (2024) 30:e14757. doi: 10.1111/cns.14757. PMID: 38747078 PMC11094579

[10] Wang J Xu L Ge Q Xue L Liu Y Wang C . EEG microstate changes during hyperbaric oxygen therapy in patients with chronic disorders of consciousness. Front Neurosci. (2023) 17:1145065. doi: 10.3389/fnins.2023.1145065. PMID: 37123360 PMC10130513

[11] Matsumoto-Miyazaki J Asano Y Takei H Ikegame Y Shinoda J . Acupuncture for chronic constipation in patients with chronic disorders of consciousness after severe traumatic brain injury. Med Acupunct. (2019) 31:218–23. doi: 10.1089/acu.2019.1361. PMID: 31456867 PMC6709722

[12] Thibaut A Schiff N Giacino J Laureys S Gosseries O . Therapeutic interventions in patients with prolonged disorders of consciousness. Lancet Neurol. (2019) 18:600–14. doi: 10.1016/s1474-4422(19)30031-6. PMID: 31003899

[13] Pitcher D Parkin B Walsh V . Transcranial magnetic stimulation and the understanding of behavior. Annu Rev Psychol. (2021) 72:97–121. doi: 10.1146/annurev-psych-081120-013144. PMID: 33095690

[14] Lefaucheur JP Aleman A Baeken C Benninger DH Brunelin J Di Lazzaro V . Evidence-based guidelines on the therapeutic use of repetitive transcranial magnetic stimulation (rTMS): an update, (2014-2018). Clin Neurophysiol. (2020) 131:474–528. doi: 10.1016/j.clinph.2019.11.002. PMID: 31901449

[15] Sharon O Fahoum F Nir Y . Transcutaneous vagus nerve stimulation in humans induces pupil dilation and attenuates alpha oscillations. J Neurosci. (2021) 41:320–30. doi: 10.1523/jneurosci.1361-20.2020. PMID: 33214317 PMC7810665

[16] Hawco C Voineskos AN Steeves JKE Dickie EW Viviano JD Downar J . Spread of activity following TMS is related to intrinsic resting connectivity to the salience network: a concurrent TMS-fMRI study. Cortex. (2018) 108:160–72. doi: 10.1016/j.cortex.2018.07.010. PMID: 30195825

[17] Dufor T Lohof AM Sherrard RM . Magnetic stimulation as a therapeutic approach for brain modulation and repair: underlying molecular and cellular mechanisms. Int J Mol Sci. (2023) 24(22). doi: 10.3390/ijms242216456. PMID: 38003643 PMC10671429

[18] Wu Y Li Z Qu R Wang Y Li Z Wang L . Electroencephalogram-based brain connectivity analysis in prolonged disorders of consciousness. Neural Plast. (2023) 2023:4142053. doi: 10.1155/2023/4142053. PMID: 37113750 PMC10129427

[19] Naro A Russo M Leo A Bramanti P Quartarone A Calabrò RS . A single session of repetitive transcranial magnetic stimulation over the dorsolateral prefrontal cortex in patients with unresponsive wakefulness syndrome: preliminary results. Neurorehabil. Neural Repair. (2015) 29:603–13. doi: 10.1177/1545968314562114. PMID: 25539781

[20] Lehembre R Marie-Aurélie B Vanhaudenhuyse A Chatelle C Cologan V Leclercq Y . Resting-state EEG study of comatose patients: a connectivity and frequency analysis to find differences between vegetative and minimally conscious states. Funct Neurol. (2012) 27:41–7. PMC3812750 22687166

[21] Schnakers C Ledoux D Majerus S Damas P Damas F Lambermont B . Diagnostic and prognostic use of bispectral index in coma, vegetative state and related disorders. Brain Inj. (2008) 22:926–31. doi: 10.1080/02699050802530565. PMID: 19005884

[22] Kondziella D Bender A Diserens K Van Erp W Estraneo A Formisano R . European Academy of Neurology guideline on the diagnosis of coma and other disorders of consciousness. Eur J Neurol. (2020) 27:741–56. doi: 10.1111/ene.14151. PMID: 32090418

[23] Teasdale G Jennett B . Assessment of coma and impaired consciousness. A practical scale. Lancet. (1974) 2:81–4. doi: 10.1016/s0140-6736(74)91639-0. PMID: 4136544

[24] Kalmar K Giacino JT . The JFK coma recovery scale--revised. Neuropsychol. Rehabil. (2005) 15:454–60. doi: 10.1080/09602010443000425. PMID: 16350986

[25] Synek VM . Prognostically important EEG coma patterns in diffuse anoxic and traumatic encephalopathies in adults. J Clin Neurophysiol. (1988) 5:161–74. doi: 10.1097/00004691-198804000-00003. PMID: 3074973

[26] Greenberg RP Newlon PG Hyatt MS Narayan RK Becker DP . Prognostic implications of early multimodality evoked potentials in severely head-injured patients. A prospective study. J Neurosurg. (1981) 55:227–36. doi: 10.3171/jns.1981.55.2.0227. PMID: 7252546

[27] Xu C Yuan Z Chen Z Liao Z Li S Feng Y . Perturbational complexity index in assessing responsiveness to rTMS treatment in patients with disorders of consciousness: a cross-over randomized controlled trial study. J NeuroEng. Rehabil. (2024) 21:167. doi: 10.1186/s12984-024-01455-1. PMID: 39300529 PMC11411826

[28] Spampinato DA Casula EP Koch G . The cerebellum and the motor cortex: multiple networks controlling multiple aspects of behavior. Neuroscientist. (2024) 30:723–43. doi: 10.1177/10738584231189435. PMID: 37649430

[29] Xia X Bai Y Zhou Y Yang Y Xu R Gao X . Effects of 10 Hz repetitive transcranial magnetic stimulation of the left dorsolateral prefrontal cortex in disorders of consciousness. Front Neurol. (2017) 8:182. doi: 10.3389/fneur.2017.00182. PMID: 28515709 PMC5413493

[30] Xia X Liu Y Bai Y Liu Z Yang Y Guo Y . Long-lasting repetitive transcranial magnetic stimulation modulates electroencephalography oscillation in patients with disorders of consciousness. Neuroreport. (2017) 28:1022–9. doi: 10.1097/wnr.0000000000000886. PMID: 28902713

[31] Liu X Meng F Gao J Zhang L Zhou Z Pan G . Behavioral and resting state functional connectivity effects of high frequency rTMS on disorders of consciousness: a sham-controlled study. Front Neurol. (2018) 9:982. doi: 10.3389/fneur.2018.00982. PMID: 30519211 PMC6258881

[32] Frohlich J Toker D Monti MM . Consciousness among delta waves: a paradox? Brain. (2021) 144:2257–77. doi: 10.1093/brain/awab095. PMID: 33693596

[33] Sullivan EG Guernon A Blabas B Herrold AA Pape TL . Familiar auditory sensory training in chronic traumatic brain injury: a case study. Disabil Rehabil. (2018) 40:945–51. doi: 10.1080/09638288.2016.1277403. PMID: 28102097

[34] Louise-Bender Pape T Rosenow J Lewis G Ahmed G Walker M Guernon A . Repetitive transcranial magnetic stimulation-associated neurobehavioral gains during coma recovery. Brain Stimul. (2009) 2:22–35. doi: 10.1016/j.brs.2008.09.004. PMID: 20633400

[35] Shah-Basak P Harvey DY Parchure S Faseyitan O Sacchetti D Ahmed A . Brain-derived neurotrophic factor polymorphism influences response to single-pulse transcranial magnetic stimulation at rest. Neuromodulation. (2020). doi: 10.1111/ner.13287. PMID: 33090650 PMC8032803

[36] Dubbioso R Pellegrino G Ranieri F Di Pino G Capone F Dileone M . BDNF polymorphism and interhemispheric balance of motor cortex excitability: a preliminary study. J Neurophysiol. (2022) 127:204–12. doi: 10.1152/jn.00268.2021. PMID: 34936818

[37] Linz R Puhlmann LMC Apostolakou F Mantzou E Papassotiriou I Chrousos GP . Acute psychosocial stress increases serum BDNF levels: an antagonistic relation to cortisol but no group differences after mental training. Neuropsychopharmacology. (2019) 44:1797–804. doi: 10.1038/s41386-019-0391-y. PMID: 30991416 PMC6785147

[38] Montone RA Camilli M Del Buono MG Russo M Rinaldi R Canonico F . Brain-derived neurotrophic factor in patients with acute coronary syndrome. Transl Res. (2021) 231:39–54. doi: 10.1016/j.trsl.2020.11.006. PMID: 33221484

[39] Le Nedelec M Glue P Winter H Goulton C Broughton L Medlicott N . Acute low-dose ketamine produces a rapid and robust increase in plasma BDNF without altering brain BDNF concentrations. Drug Delivery Transl Res. (2018) 8:780–6. doi: 10.1007/s13346-017-0476-2. PMID: 29322484

[40] Wang CS Kavalali ET Monteggia LM . BDNF signaling in context: from synaptic regulation to psychiatric disorders. Cell. (2022) 185:62–76. doi: 10.1016/j.cell.2021.12.003. PMID: 34963057 PMC8741740

[41] Videtta G Squarcina L Rossetti MG Brambilla P Delvecchio G Bellani M . White matter modifications of corpus callosum in bipolar disorder: a DTI tractography review. J Affect Disord. (2023) 338:220–7. doi: 10.1016/j.jad.2023.06.012. PMID: 37301293

[42] Joseph CR . Assessing mild traumatic brain injury-associated blood-brain barrier (BBB) damage and restoration using late-phase perfusion analysis by 3D ASL MRI: implications for predicting progressive brain injury in a focused review. Int J Mol Sci. (2024) 25(22). doi: 10.3390/ijms252111522. PMID: 39519073 PMC11547134

[43] Chou YH Sundman M Ton That V Green J Trapani C . Cortical excitability and plasticity in Alzheimer's disease and mild cognitive impairment: a systematic review and meta-analysis of transcranial magnetic stimulation studies. Ageing Res Rev. (2022) 79:101660. doi: 10.1016/j.arr.2022.101660. PMID: 35680080 PMC9707650

[44] Bai Z Zhang J Fong KNK . Effects of transcranial magnetic stimulation in modulating cortical excitability in patients with stroke: a systematic review and meta-analysis. J NeuroEng. Rehabil. (2022) 19:24. doi: 10.1186/s12984-022-00999-4. PMID: 35193624 PMC8862292

