# Supplementary figures and images for: Retrospective evaluation of transcranial magnetic stimulation for enhancing arousal in patients with minimally conscious state: a single-centre study in Inner Mongolia, China
Source: Front Psychiatry. 2026 May 5;17:1757883. doi: 10.3389/fpsyt.2026.1757883 (PMC13183859; doi:10.3389/fpsyt.2026.1757883)

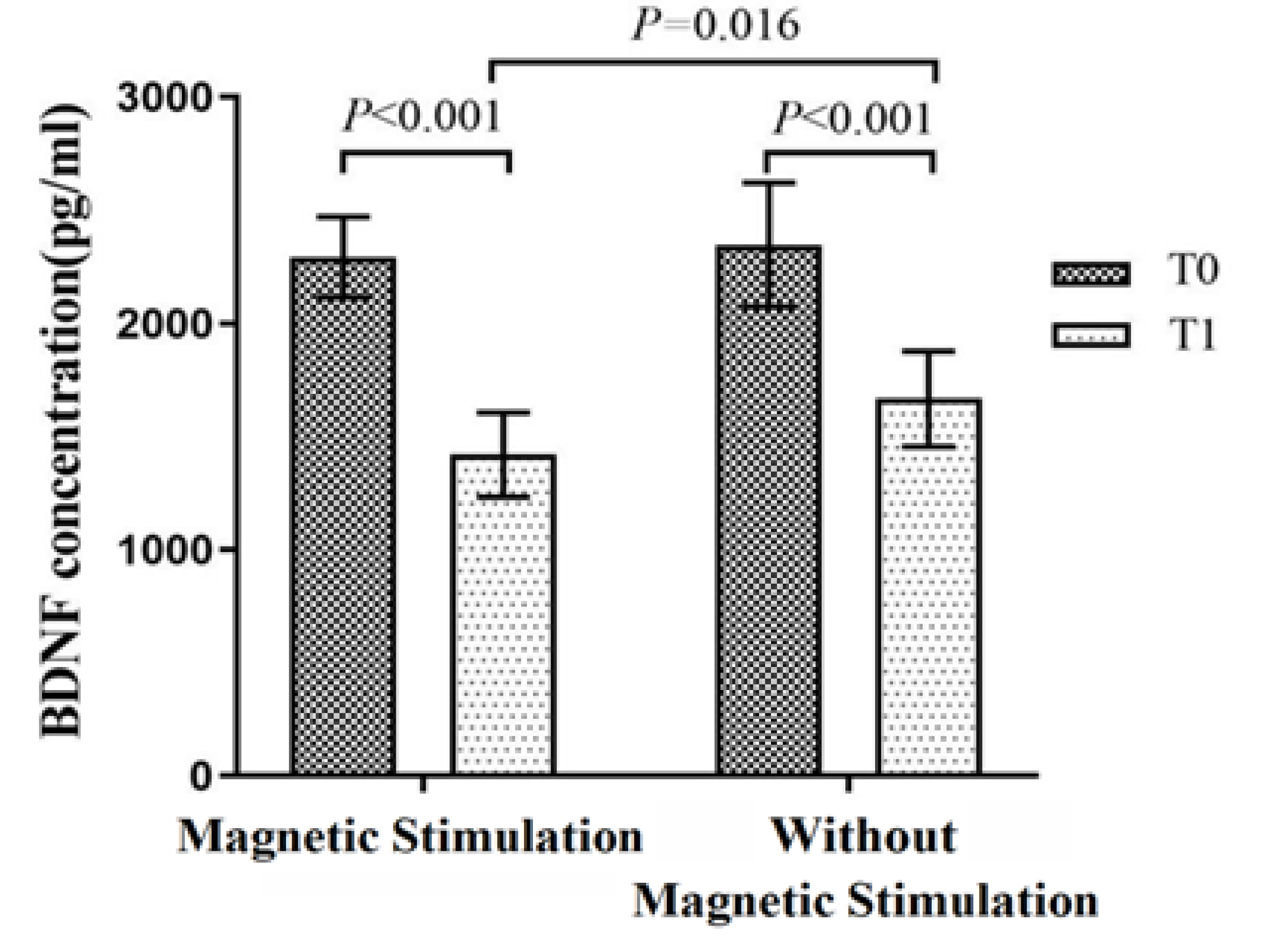

Supplement: Supplementary file 1 [file Image1.tif]

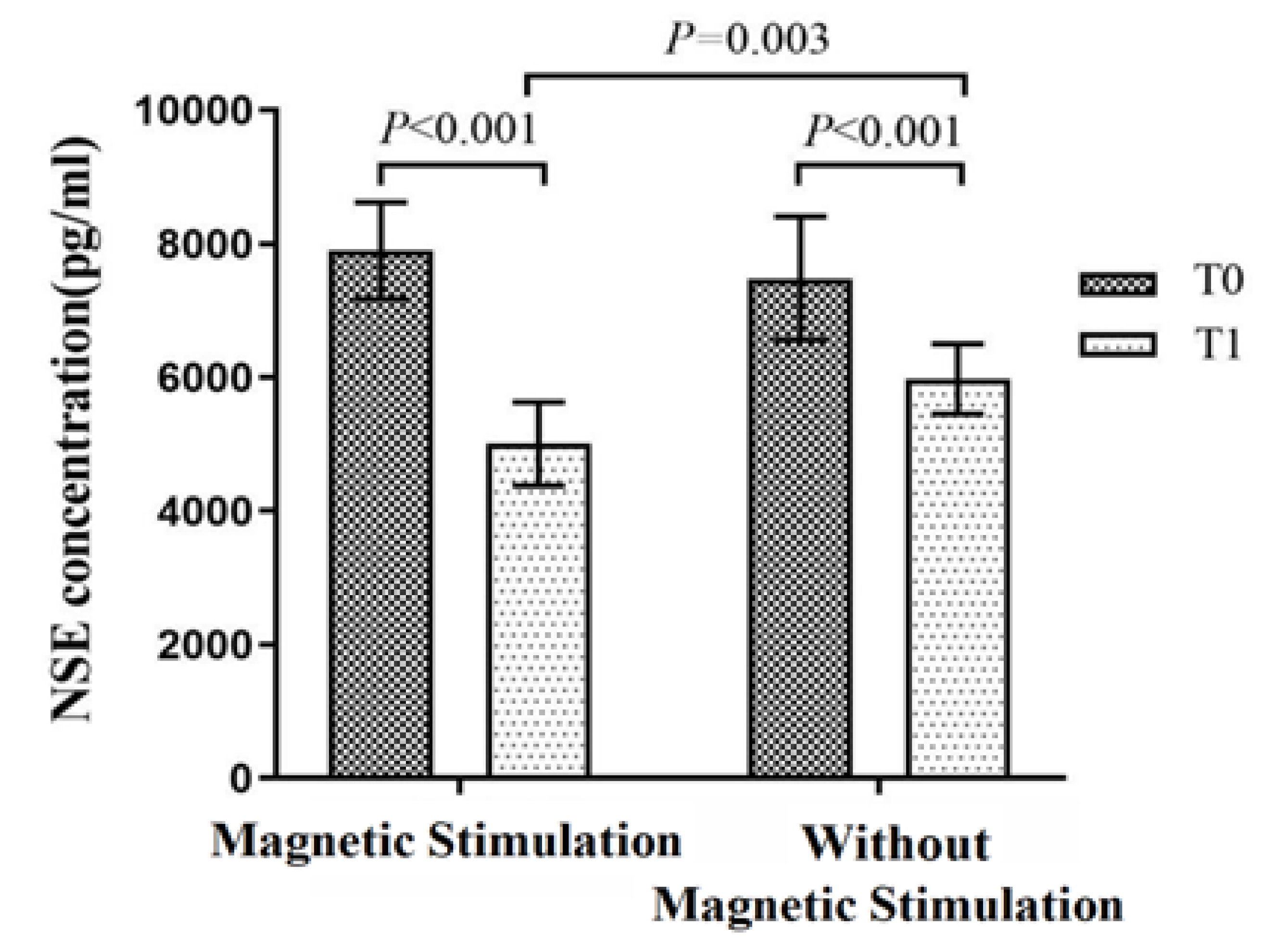

Supplement: Supplementary file 2 [file Image2.tif]

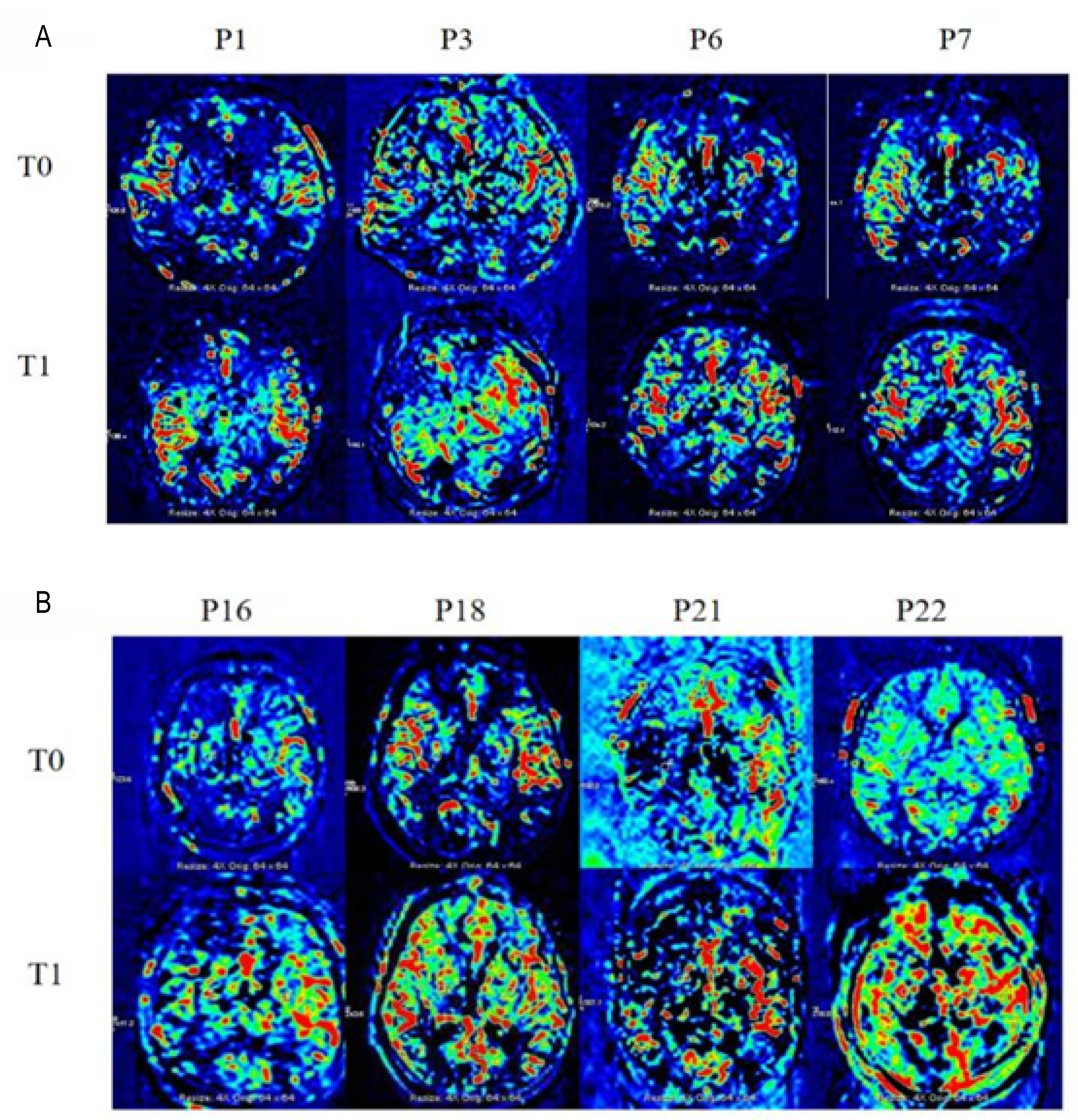

Supplement: Supplementary file 3 [file Image3.tif]

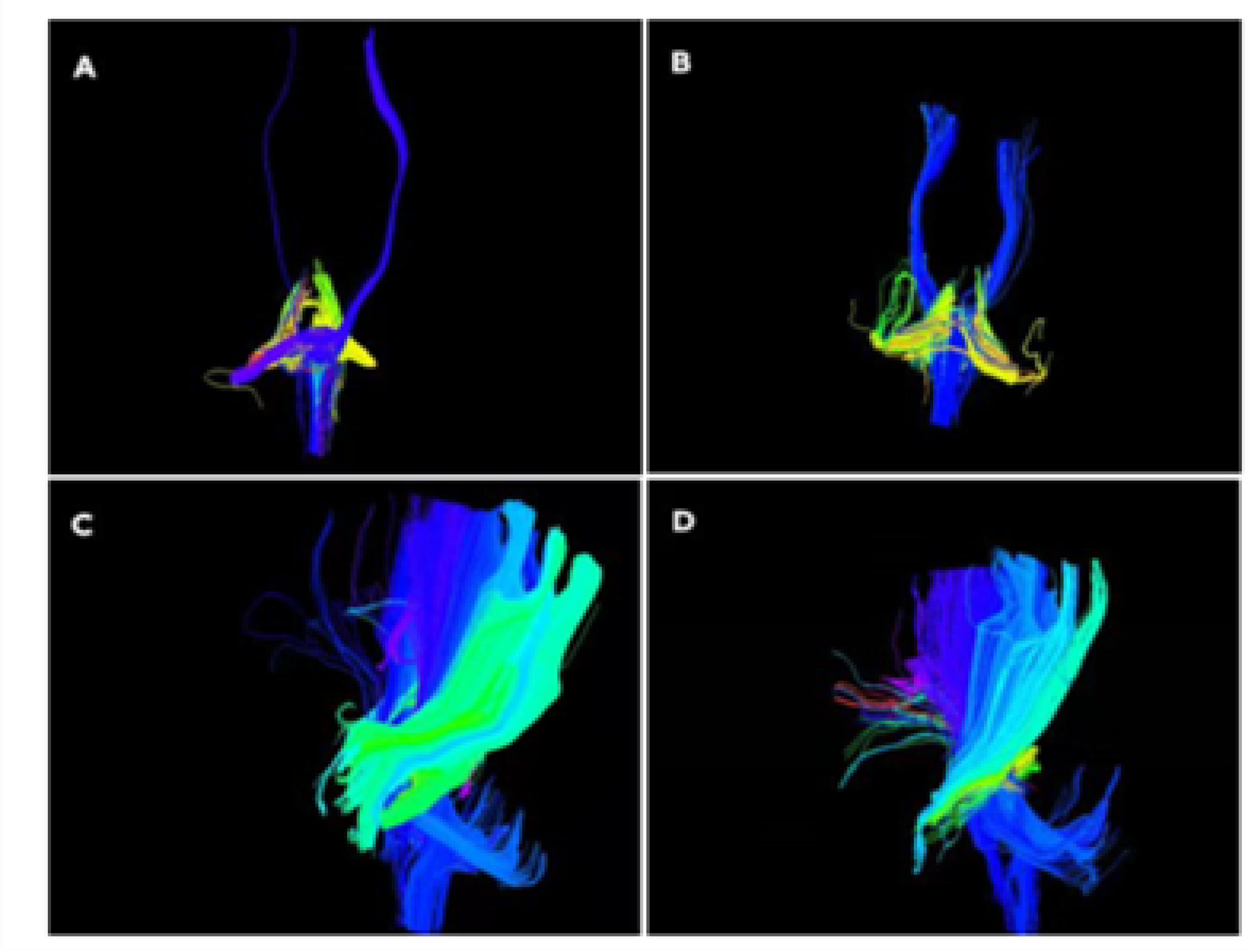

Supplement: Supplementary file 4 [file Image4.tif]
